# Magnetic Field-Enhancing Photocatalytic Reaction in Micro Optofluidic Chip Reactor

**DOI:** 10.1186/s11671-019-3153-1

**Published:** 2019-10-15

**Authors:** Hung Ji Huang, Yen Han Wang, Yuan-Fong Chou Chau, Hai-Pang Chiang, Jeffrey Chi-Sheng Wu

**Affiliations:** 1 Taiwan Instrument Research Institute, National Applied Research Laboratories, Hsinchu, Taiwan; 2 Department of Chemical Engineering, National Taiwan University, Taipei, Taiwan; 3 Centre for Advanced Material and Energy Sciences, Universiti Brunei Darussalam, Gadong, Negara Brunei Darussalam; 4 Institute of Optoelectronic Sciences, National Taiwan Ocean University, Keelung, Taiwan; 5 Institute of Physics, Academia Sinica, Taipei, Taiwan

**Keywords:** Magnetic field, Photocatalytic reactions, Micro optofluidic chip, Titanium dioxide, Ion condensation, Hot charge carriers

## Abstract

A small external magnetic field (100–1000 Oe) was demonstrated to enhance the photocatalytic degradation of methyl orange (MO) using TiO_2_ NPs in micro optofluidic chip (MOFC) reactors. The rectangular shape of the fluidic channel and TiO_2_ deposited only onto the lower glass substrate leads to a selectively enhancing photocatalytic reactions by magnetic field in specific directions. Utilizing ethyl alcohol as a scavenger presented the difference between generated hot-hole (hVB^+^) and hot-electron (eCB^−^) pathways of photocatalytic reactions. Effects of dissolved oxygen (DO) and hydroxyl ions (OH^−^) are all demonstrated in a magnetic field-enhancing photocatalytic reaction. The experimental results demonstrate great potential for practical applications utilizing low-price fixed magnets in the field of green chemistry.

## Introduction

Many methods have been suggested for improving the performance of photocatalytic reactions, such as through material modification and introducing new types of photocatalytic reactors [1–4]. Material modification or using composite materials [5–10] and plasma treatment [11–13] have also been suggested to improve photocatalytic processing efficiency. Magnetic photocatalysts have attracted considerable interest as they can be easily collected after reactions and recycled for further reuse. In some studies, the externally applied magnetic field was even demonstrated to boost the photocatalytic processing efficiency [14–20]. As a non-magnetic photocatalyst, improved processing efficiencies have also been observed for TiO_2_ under an external magnetic field. However, a remarkably strong magnetic field, up to several kOe or 1 T, was utilized for observable enhancements in photocatalytic reactions employing TiO_2_.

The external magnetic field can enhance the photocatalytic reactions by boosting carrier transport [20], reducing the recombination of light-induced hot-charge carriers [14], and forcing migration or increasing the mass transfer rate of charged chemicals (ions) in a solution (the magneto-hydrodynamic (MHD) effect) [15]. DO was also demonstrated to play an important role in magnetic field-enhancing photocatalytic reactions according to the oxygen-acceleration-near-surface (OANS) model [15–19]. Utilizing slurry bed reactors or fixed bed reactors in the reference works required magnetic field up to 0.5–1.5 T (10^4^ Oe) [14–20] to have a noticeable enhancement on photocatalytic reactions.

In the present study, applying a small magnetic field (~ 100 Oe) is demonstrated to boost the photocatalytic degradation of methyl orange within a micro optofluidic chip reactor. Modern green chemistry is looking for low power consumption, small occupation, and low waste. The photocatalytic reactions boosting by small magnetic field (easily provided from fixed magnets) demonstrated a great advance in green chemistry.

## Method

**Fig. 1:**
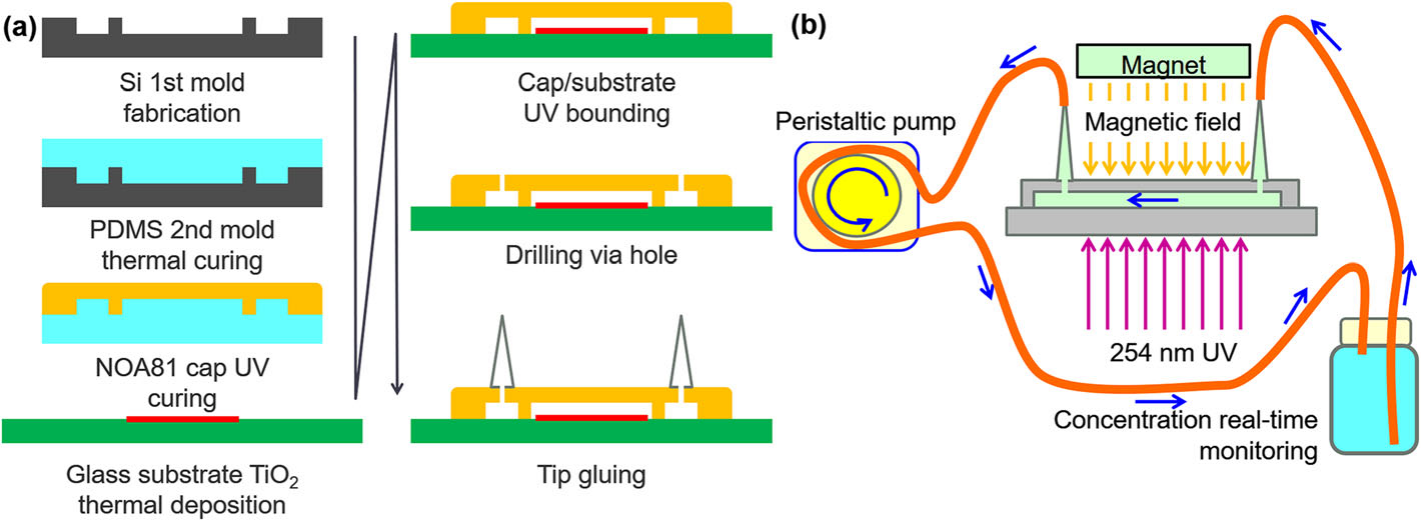
Schematics of **a** the chip fabrication process and **b** the experimental setup

A MOFC reactor with a polymer cap (UV-curable Norland Optical Adhesive; NOA81) [1] was placed under a magnetic field in various direction. The MOFC reactor was fabricated following the procedure shown in Fig. 1a.

TiO_2_ NPs (Degussa, P25) were deposited with a 0.5 mL gel solution (0.1 g P25 TiO_2_ NPs in 100 mL DI water) on the surface area of a glass microscope slide that was not covered by tape. After 48 h of slow-drying in air (covered under a plastic petri dish), the tape was removed. Finally, DI water was used to wash off the unfixed TiO_2_ NPs and the slide was dried under flowing N_2_ gas. The glass substrates coated with P25 TiO_2_ NPs (~ 0.5 mg in 1.5 × 2.5 cm^2^) were then ready for sealing to the NOA81 polymer upper cap (main body of the microfluidic chip).

A silicon first mold was produced via inductively coupled plasma deep dry etching after preparation of a SiO_2_ hard mask. The polydimethylsiloxane (PDMS) mold was heated to 75 °C for 20 min to cure. The NOA81 polymer cap was fabricated using the PDMS mold under UV light illumination. The NOA81 polymer cap layer was quickly stripped down from the PDMS mold and fixed on a glass slide with an extra UV light illumination. Next, two holes were drilled through the NOA81-capped layer. Two tips were glued using NOA81 and became inlet and outlet of the micro reaction chamber.

NOA81 UV glue (Norland Optical Adhesive 81) is a single component liquid adhesive that cures in seconds into a tough, hard polymer when exposed to ultraviolet light. Interestingly, it can softly cure in a PDMS mold under limited exposure to UV light. The surface adjacent to the PDMS mold surface can remain adhesive to glass. Therefore, the NOA81 upper cap that cured in the PDMS mode could be easily fixed to the glass substrate under further UV light illumination. The deposited P25 TiO2 NPs adhere to the micro optofluidic chip without to the need for additional plasma treatments that are typically needed in the fabrication of microfluidic chips employing a PDMS top cover. This is beneficial for simplifying the repeatable experimental process because plasma treatment will increase the oxygen vacancies on the surface and alter the material properties of TiO2 NPs.

Figure 1b shows the experimental setup of the magnetic field-enhanced photocatalytic reaction. The closed-loop included a micro optofluidic chip reactor, soft tubing (Tygon E-3603 tubing, Saint-Gobain Performance Plastics, USA), and a glass bottle. A peristaltic pump drove the test solution to circulate in the closed-loop. A home-made system, using 468 nm light absorption, measured the minute-by-minute concentration of the methyl orange test solution flowing through the glass bottle. A 4-watt low-pressure mercury lamp supplied 254 nm UV light to activate the deposited commercial P25 TiO_2_ nanoparticles (NPs). The original concentration of the 20 mL test solution was 5 μM. In all experiments, an aluminum reflector served as a light reflector to maintain the illumination intensity on the deposited TiO_2_ NPs and shield the experimenters.

**Fig. 2:**
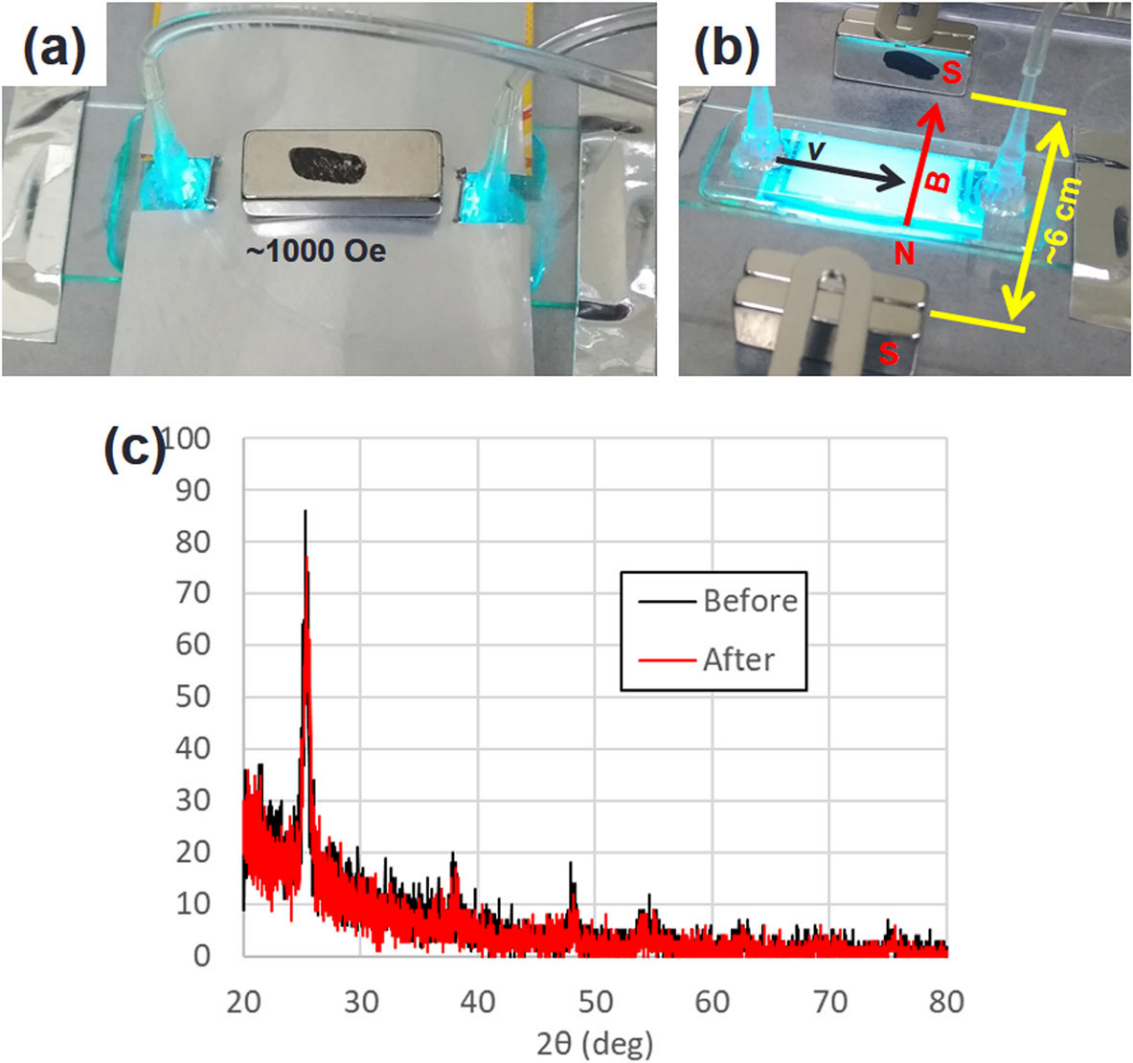
Experimental setup and effects of applying a magnetic field to enhance a photocatalytic reaction. Various arrangements of the neodymium magnets to provide **a** a normal magnetic (NM) field and **b** a lateral magnetic (LM) field. **c** X-ray diffraction pattern before and after UV light treatment in a ~ 1000 Oe magnetic field

Rare earth metal neodymium magnets (25 × 10 × 5 mm), containing an alloy of Nd, Fe, and B, were purchased from a local bookstore and they provided static magnetic fields of up to 3000 Oe. They were arranged to supply a magnetic field normal or parallel to the TiO_2_ layer (Fig. 2a, b). The high-intensity magnetic field areas near the pores of the magnets were not used in this study. The normally arranged magnet supplied a vertical magnetic field of around 1000 Oe when placed about 5 mm above the photocatalytic reaction area. The laterally arranged magnets (photocatalytic area between the magnets, distance between magnets ~ 6 cm) supplied a homogeneous parallel magnetic field (< 5% deviation) in the area of reaction. The magnetic field in the *x*-direction (parallel to the flow direction in the microfluidic channel) was less than 5% of that in the *y*-direction in the reaction area. Thus, we could focus on the effect of the magnetic field in the *y*-direction (perpendicular to the flow direction in microfluidic channel). The deposited P25 TiO_2_ NPs were stable under UV light illumination and to the magnetic field. The X-ray diffraction pattern of the deposited P25 TiO_2_ NPs presented no observable differences before and after 3 h UV light illumination under a ~ 1000 Oe magnetic field, as shown in Fig. 2c.

## Results and Discussion

**Fig. 3:**
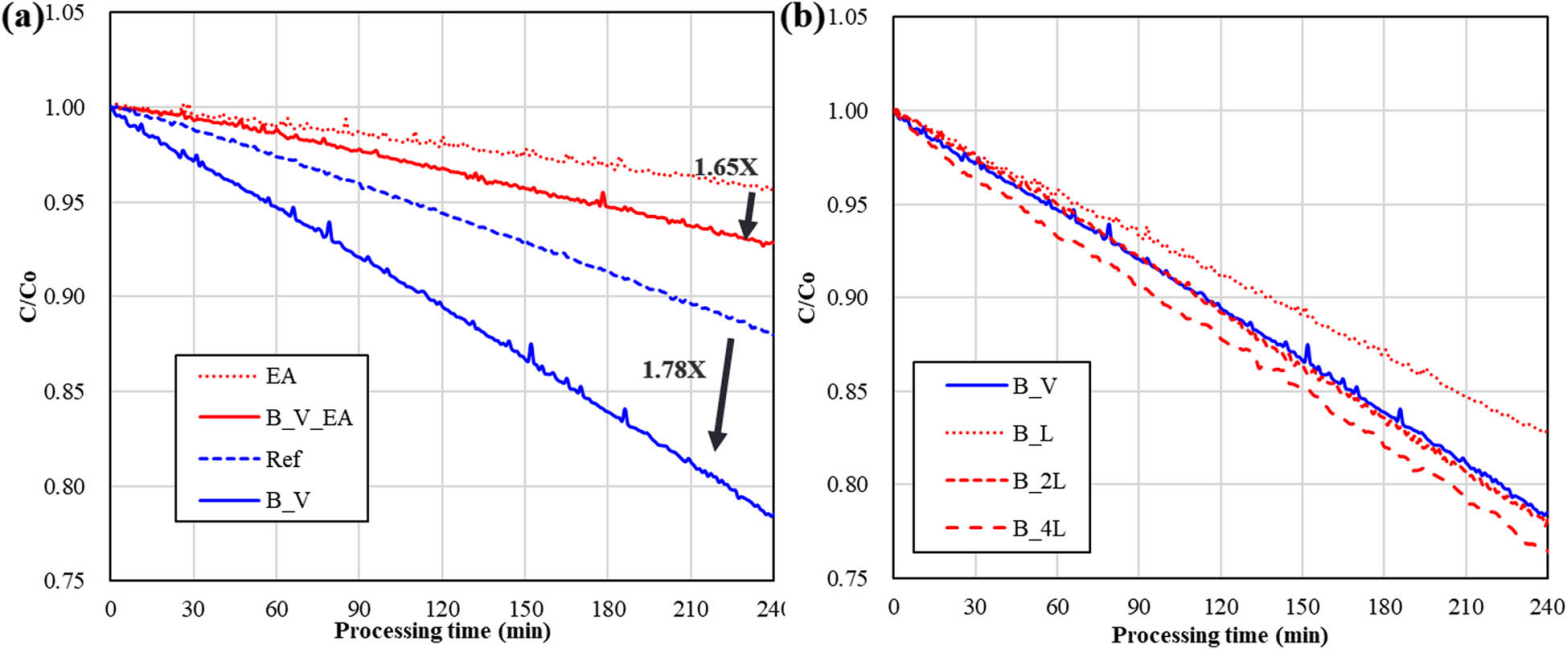
Photocatalytic degradation of MO under the application of (**a**) a vertical magnetic field with and without EA and (**b**) various magnetic fields

The results of the 240 min (4 h) long experiments show that applying an external vertical magnetic field (B), as shown in Figs. 2a and 6a, to the photocatalytic degradation of MO in a MOFC reactor increased the C/Co decay rate (Fig. 3). The photocatalytic degradation of (5 μM starting concentration for all experiments) proceeds in the following steps [17, 21]:

1. TiO_2_ + hν → TiO_2_ (hVB^+^) + TiO_2_ (eCB^−^)
2. TiO_2_ (hVB^+^) + H_2_O → TiO_2_ + H^+^ + OH^−^
3. TiO_2_ (hVB^+^) + OH^−^ → TiO_2_ + *OH
4. MO + *OH → degradation products
5. TiO_2_ (eCB^−^) + O_2_ → TiO_2_ + *O_2_^−^
*O_2_^−^ + H^+^ → *HO_2_
7. MO + *HO_2_^−^ → degradation products

The experimental results show that the NM field can increase the total degraded ratio of MO 1.78-fold, (1-B_V)/(1-ref). In the experiments containing ethyl alcohol (EA) additive, within a processing time of 4 h, the external magnetic field increased the total degraded ratio of MO in the eCB^−^ pathway, (1-B_V_EA)/(1-EA).

The effect of applying lateral magnetic (LM) field (Fig. 2b) was also studied. The magnitude of the LM field was varied using different combinations of magnets. Pairs of magnets provide various LM field that is parallel to the plane of the TiO_2_ deposition. As shown in Fig. 3b, a pair of neodymium magnets (B-L) provides a magnetic field up to 90 ± 5 Oe. Four pairs and two pairs of magnets (B-4 L and B-2 L in Fig. 3b, respectively) were also used to study the effects of increased magnetic field strength on the photocatalytic degradation of MO. In both cases, the degradation efficiency was increased relative to that produced using the vertically applied magnetic field (B-V, shown in Fig. 3b). Note that the magnitude of the vertically applied magnetic field was ~ 1000 Oe, which was much stronger than that in the lateral arrangement. Therefore, the enhancement in the photocatalytic degradation of MO due to the application of a LM field was better than that due to the application of the NM field.

**Fig. 4:**
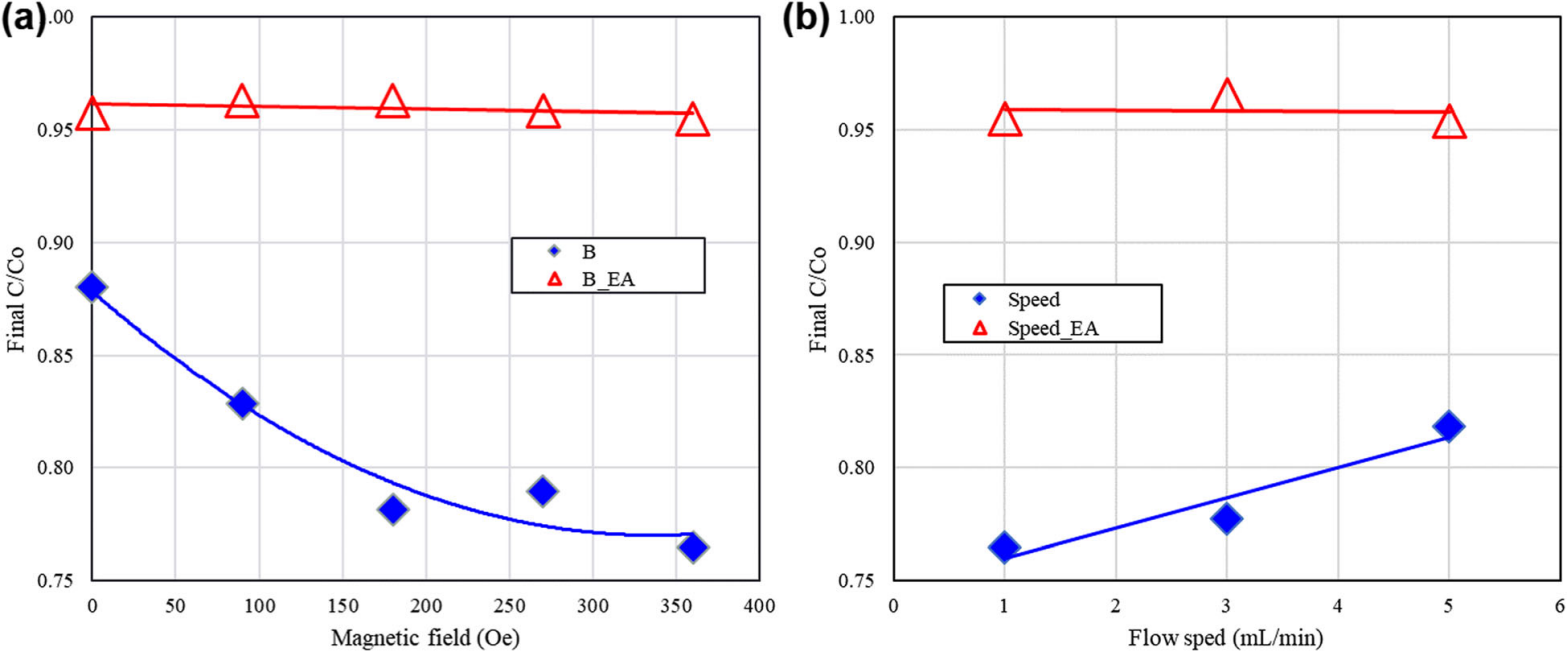
Experimental results. **a** Photocatalytic degradation of MO from the application of various magnetic fields with and without EA. **b** Effects of flow speed in the magnetic field-enhanced photocatalytic reaction in the micro optofluidic chip reactor

To further understand the effects of the magnetic field on the path of the chemical reaction, we examined the photocatalytic degradation of MO with and without EA under LM field conditions of different magnitudes (Fig. 4a). The 0.16 mL EA was added to the 20 mL test solution. EA served as scavenger [22, 23] of hot holes generated in the TiO_2_ NPs under 254 nm light illumination. The laterally applied magnetic fields positively enhanced the photocatalytic degradation of MO without EA. However, in the experiments with EA, no obvious difference was observed from increasing the LM field strength. The added EA functions as a scavenger of light-induced hot holes (hVB^+^). The reaction steps 2, 3, and 6 were suppressed in the experiments containing EA. The experimental results in Fig. 4a show that the photocatalytic reaction steps 5–7 of the eCB^−^ reaction path are not affected by the LM field.

**Fig. 5:**
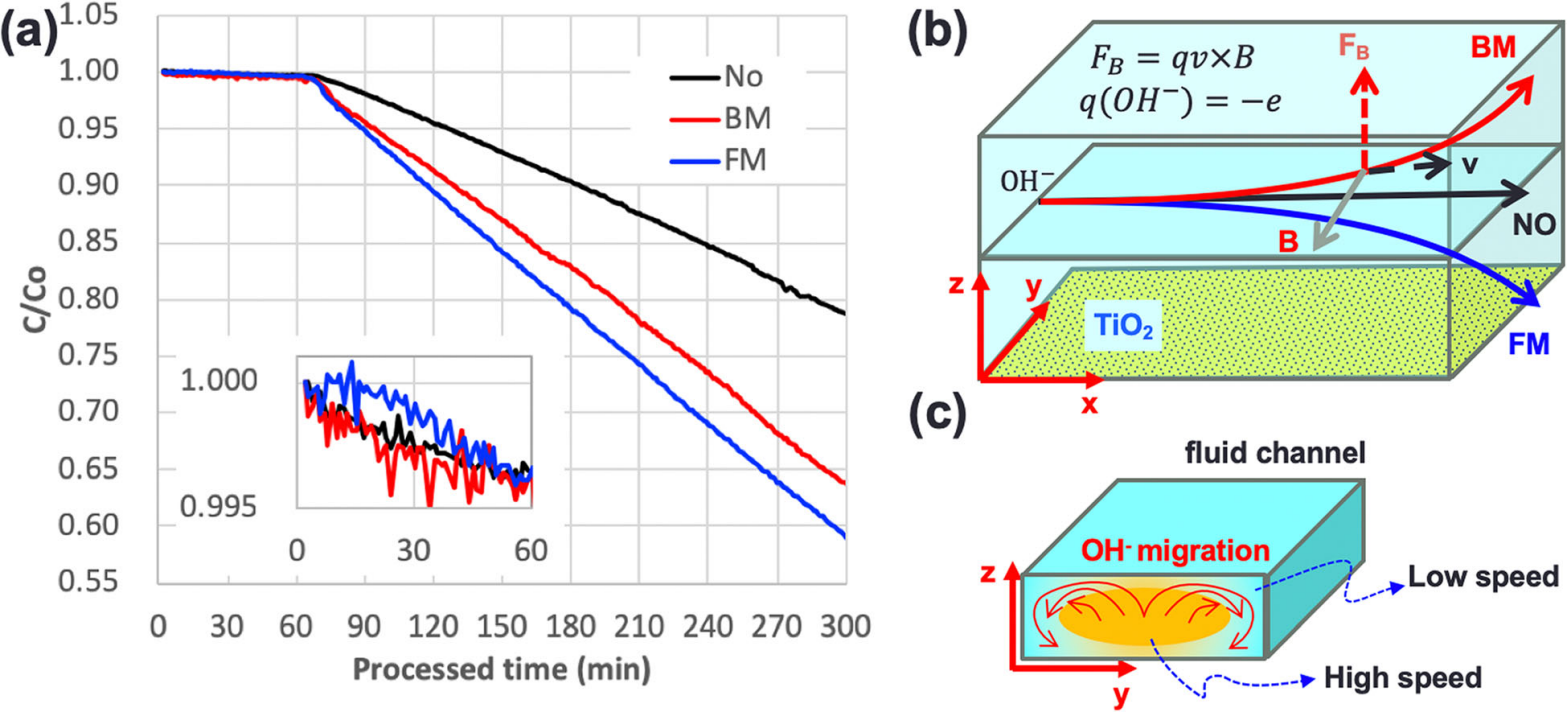
Magnetic field effects on dark absorption and OH^−^ migration. **a** Photocatalytic degradation of MO under the application of BM and FM. **b** Schematic of the magnetic field-induced migration of OH^−^ in the micro optofluidic chip reactor. **c** Schematics of the OH^−^ migration by electrostatic force in the fluidic channel in BM case

To further understand the effect of the magnetic field direction and dark adsorption of MO molecules on the photocatalytic reaction, additional experiments were performed using LM fields in opposite directions, as shown in Fig. 5a. The embedded picture in Fig. 5a presents the dark adsorption of MO by the deposited P25 TiO_2_ NPs without the illumination of UV light in the first hour of the experiment. The magnetic field in opposite directions (BM, FM) and the experiment with no magnetic field (No) provided similar results in the dark adsorption step. After the 1 h dark adsorption, the UV light was turned on and MO photocatalytic degradation commenced. The MO photocatalytic degradations with LM fields had higher processing efficiency than that with no magnetic field (No), as shown in Fig. 5.

**Fig. 6:**
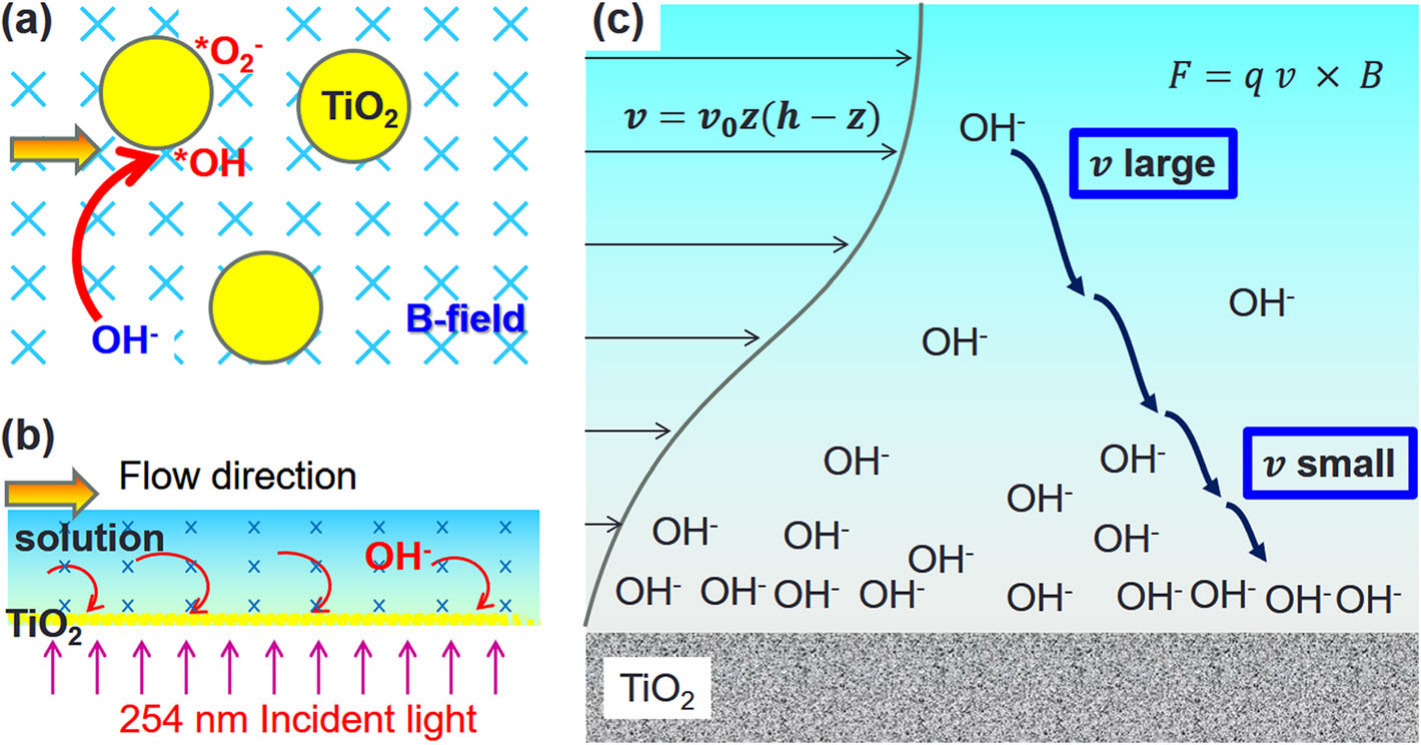
**a** Photocatalytic degradation of MO under various magnetic fields with and without EA. **b** Effects of flow speed in the magnetic field-enhanced photocatalytic reaction in the micro optofluidic chip reactor. **c**. Effect of ion-condensation of OH in microfluidics

From the experimental results of Figs. 4 and 5, it is believed that the forced moving of OH^−^ (velocity *v* and charge *q = −e*) by magnetic force (*F*_*B*_ *= qv × B*) enhances the photocatalytic reaction efficiency. According to the Hagen–Poiseuille equation, the flow speed of the plane Poiseuille flow at various positions (*z*) related to the sidewall of the fluidic channel can be simply described as *v*_*z*_ *= v*_*0*_*z*(*h − z*) [24]; herein, for typical microfluidics, *v*_*z*_ = 0 at the top (*z = h*) and bottom walls (*z = 0*) act as the no-slip boundary condition at the smallest channel width axis, as shown in Fig. 6. Therefore, *v*_max_ *= v*_*0*_ at the half-height of the microflow channel (*z = h/2*). Upon application of an external magnetic field, the external magnetic force pushes hydroxyl ion (OH^−^) from the high-speed layer to accumulate in the low-speed layer near the deposited TiO_2_. The OH^−^ concentration at the channel boundary (*z = 0, h*) increases with the increasing external magnetic field and can be named as “ion condensation.” In statistical mechanics, the chemical potential of OH^−^ in a test solution is *μ = k*_*B*_ *T* log(*n/n*_*Q*_) [25], where *k*_*B*_ is the Boltzmann constant, *n* is the concentration of OH^−^, and *n*_*Q*_ = [(*M k*_*B*_ *T/2πℏ*^*2*^)]^3/2^ is the quantum concentration of OH^−^ at temperature *T*. *M* is mass of OH^−^. *ℏ* is reduced Planck constant. Therefore, the chemical potentials μ_B_ *= k*_*B*_ *T* log(*n/n*_*Q*_) of OH^−^ at *z = 0,* and *h* are increased by the external field B.

In the BM case, the magnetic field forces the OH^−^ ions to move out from the high-flow-speed center area and to the low-flow-speed upper part of no deposited TiO_2_. The accumulated OH^−^ ions electrically expel each other to diffuse in the low-flow-speed area near the wall of the fluidic channel, as shown in Fig. 5c. The concentration of OH^−^ adjacent to the deposited TiO_2_ thus gradually increases. This indirectly improved mass transfer rate of OH^−^ to the deposited TiO_2_ in the BM case processes the photocatalytic reactions in higher efficiency compared with that of no applying magnetic field.

Figure 4b shows the effect of the flow speed on the magnetic field-enhanced photocatalytic reaction in a micro optofluidic chip reactor. The results show that an increase in the flow speed or the traveling speed of the charged ions (*v*) results in a decrease in the photocatalytic degradation efficiency and a decrease in the residence time of the material traveling in the fluidic chip. They lead to a significant decrease in the generation rate of *OH. Overall, an increase in the flow speed results in a small, but still observable, decrease in the hot-electron path of the photocatalytic reaction.

In the NM field case (Fig. 3a), the OH^−^ is forced to move circularly on the plane parallel to the deposited TiO_2_ layer. This also increases the mass transfer rate in the fluid and the photocatalytic processing efficiency, as shown in Fig. 4. However, the addition of EA cannot suppress the hot-hole path of the MO photocatalytic degradation pathway in the microfluid. The large magnetic field (~ 1000 Oe) can enhance the photocatalytic reactions by a complex mechanism beyond the migration or condensation of OH^−^ in the microfluids. This means that a giant magnetic field can partially overcome the effect by adding the hot-hole scavenger (EA).

**Fig. 7:**
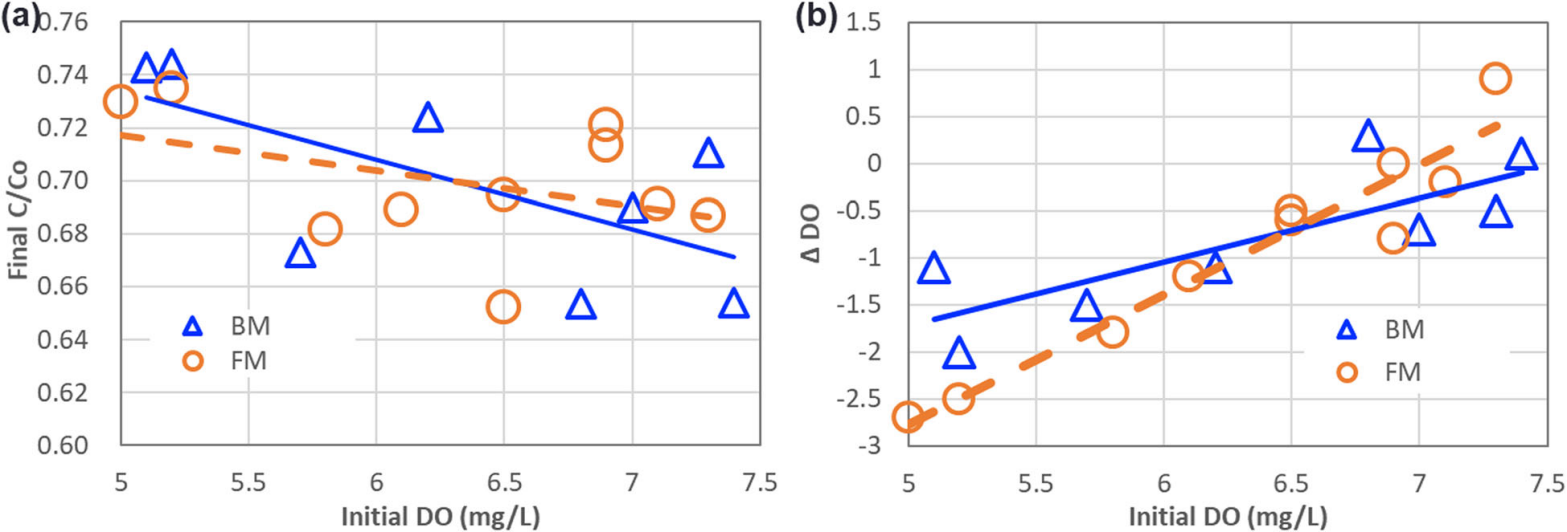
Magnetic field-affected photocatalytic degradation of MO under the application of BM and FM with various concentration of dissolved oxygen. **a** Final C/Co and **b** difference on (used) dissolved oxygen before and after process

In the reference works, the OANS effect [16–19] was suggested to be responsible for the magnetic field effect in enhancing photocatalytic reactions. An additional experiment in the magnetic field-assisted photocatalytic reactions is also processed regarding the dissolved oxygen following the same experimental procedure in Fig. 5. The DO values were measured using a DO meter (DO-5510, Lutron Electronic Enterprise Co. Ltd.). The original DO level was altered with bubbling air into the test solution. The final C/Co is roughly decreased with the increase of DO concentration. Therefore, the processing efficiency of the magnetic photocatalytic reactions is positively depending on the initial DO. The results also show that, as shown in Fig. 7b, the negative difference between dissolved oxygen before and after means generation of oxygen is also happening in the process. This might come from the photocatalytic generation of oxygen.

The OANS effect suggested that the oxygen molecules can form complex chemicals with dye molecules and attract to the surface of the photocatalyst under external light illumination and magnetic field. This leads to enhancement on magnetic photocatalytic reaction. However, generation of oxygen will also consume the induced hVB^+^. Therefore, OANS effect and photocatalytic generation of oxygen result in low photocatalytic MO degradation efficiency when initial DO concentration is low in the test solution.

## Conclusion

The effects of a small magnetic field (100–1000 Oe) on a photocatalytic reaction using TiO_2_ NPs were resolved by applying various magnetic fields on micro optofluidic chip reactors. The rectangular fluidic channel and TiO_2_ deposited only onto the substrate surface that leads to studies with magnetic field in specific directions. Utilizing EA as a scavenger additive allowed for focused studies on the hot-hole and hot-electron photocatalytic reaction pathways. A small laterally arranged magnetic field mainly affects the migration of ions in the microfluids. Concentration of the dissolved oxygen (DO) also strongly affects the processing efficiency of the magnetic field-affected photocatalytic reactions. Neodymium magnets can supply a constant magnetic field and allow for photocatalytic reaction enhancements without additional energy inputs. Therefore, our results confirm that the application of a smaller static magnetic field can enhance photocatalytic reactions, thus returning this phenomenon within the tenants of green chemistry.

## Data Availability

All data generated or analyzed during this study are included in this published article.

## Abbreviations

C/Co: Reserved ratio of target reactants, that is MO in this paper
DO: Dissolved oxygen
EA: Ethyl alcohol
eCB^−^: Hot electrons in conduction band
hVB^+^: Hot holes in valence band
LM: Lateral magnetic
MHD: Magneto-hydrodynamic
MO: Methyl orange
MOFC: Micro optofluidic chip
NM: Normal magnetic
NPs: Nanoparticles
OANS: Oxygen-acceleration-near-surface
Oe: Oersted, unit of the auxiliary magnetic field H in the centimeter–gram–second system of units (CGS)
OH^−^: Hydroxyl ion
QHCs: Quantum hot-charge carriers
T: Tesla (symbol T) is a derived unit of the magnetic field strength (also, magnetic flux density) in the International System of Units.
UV: Ultraviolet
